# Evaluation of the feasibility and acceptability of the ‘Care for Stroke’ intervention in India, a smartphone-enabled, carer-supported, educational intervention for management of disability following stroke

**DOI:** 10.1136/bmjopen-2015-009243

**Published:** 2016-02-02

**Authors:** K Sureshkumar, GVS Murthy, S Natarajan, C Naveen, S Goenka, H Kuper

**Affiliations:** 1 Department of Clinical Research, International Centre for Evidence in Disability, London School of Hygiene and Tropical Medicine, London, UK; 2 TS Srinivasan Institute of Neurological Sciences, VHS Hospital, Chennai, Tamil Nadu, India; 3 Indian Institute of Public Health (Pubic Health Foundation of India) Hyderabad, Amar Cooperative Society, Hyderabad, Telangana, India; 4 Indian Institute of Public Health (Public Health Foundation of India), Delhi, Haryana, India

**Keywords:** PUBLIC HEALTH, REHABILITATION MEDICINE, GERIATRIC MEDICINE

## Abstract

**Objectives:**

(1) To identify operational issues encountered by study participants in using the ‘Care for Stroke’ intervention; (2) to evaluate the feasibility and acceptability of the intervention.

**Design:**

Mixed-methods research design.

**Setting:**

Participant's home. Participants were selected from a tertiary hospital in Chennai, South India.

**Participants:**

Sixty stroke survivors treated and discharged from the hospital, and their caregivers.

**Intervention:**

‘Care for Stroke’ is a smartphone-enabled, educational intervention for management of physical disabilities following stroke. It is delivered through a web-based, smartphone-enabled application. It includes inputs from stroke rehabilitation experts in a digitised format.

**Methods:**

Evaluation of the intervention was completed in two phases. In the first phase, the preliminary intervention was field-tested with 30 stroke survivors for 2 weeks. In the second phase, the finalised intervention was provided to a further 30 stroke survivors to be used in their homes with support from their carers for 4 weeks.

**Primary and secondary outcome measures:**

Primary outcomes: (1) operational difficulties in using the intervention; (2) feasibility and acceptability of the intervention in an Indian setting. Disability and dependency were assessed as secondary outcomes.

**Results:**

Field-testing identified operational difficulties related to connectivity, video-streaming, picture clarity, quality of videos, and functionality of the application. The intervention was reviewed, revised and finalised before pilot-testing. Findings from the pilot-testing showed that the ‘Care for Stroke’ intervention was feasible and acceptable. Over 90% (n=27) of the study participants felt that the intervention was relevant, comprehensible and useful. Over 96% (n=29) of the stroke survivors and all the caregivers (100%, n=30) rated the intervention as excellent and very useful. These findings were supported by qualitative interviews.

**Conclusions:**

Evaluation indicated that the ‘Care for Stroke’ intervention was feasible and acceptable in an Indian context. An assessment of effectiveness is now warranted.

**Strengths and limitations of this study**

- A phased approach to the development and evaluation of the intervention helped refine the intervention.
- Mixed research methods were used for evaluation of the intervention.
- Recruitment of participants from only one centre.
- Stringent inclusion criteria for participant recruitment.

## Background

Each year, about 15 million people suffer stroke globally. One-third of stroke survivors experience permanent disability.^1^ Increased population aging and the rising prevalence of risk factors for stroke will further increase the number of people living with stroke-related disabilities.^2^ Projections by the WHO show that the disability-adjusted life years lost to stroke will rise from 38 million in 1990 to 61 million by 2020.^1^ These projections imply an overwhelming global demand for stroke rehabilitation services.^3^ This is especially true in low- and middle-income countries (LMICs), which bear a substantial amount of the global burden of stroke^4^ yet have few rehabilitation services available.

The high burden of stroke but lack of rehabilitation services creates the need to develop and evaluate innovative strategies such as the use of mobile phones or smartphone-based applications for provision of healthcare services.^5^ These mobile health (Mhealth) strategies capitalise on the core functionalities of a mobile or smartphone and are strongly recommended by the WHO for bridging the gaps in accessibility to health services globally.^6^ This was the rationale for developing ‘Care for Stroke’, which is a web-based, smartphone-enabled, caregiver-supported, educational intervention for management of physical disabilities following stroke. This Mhealth intervention draws on the principles of both medical sciences and information technology to address the gaps in access to stroke rehabilitation services for stroke survivors in a systematic way, as recommended by the Medical Research Council.^7^ ^8^ The intervention has been developed with a specific focus on LMICs, where the resources available for rehabilitation are often very limited. To our knowledge, there are no stroke rehabilitation interventions enabled through Mhealth platforms that are available and relevant to LMICs, such as India, where the resources for rehabilitation are limited and the unmet needs of stroke survivors are substantial.^9^ Therefore, it was decided to evaluate this newly developed rehabilitation intervention in an Indian context.

The research study protocol which describes the participatory development of the intervention is available elsewhere.^10^ The present paper describes the field-testing and pilot-testing of the intervention. The purpose of field-testing was to provide the newly developed intervention to stroke survivors and their caregivers and assess any initial operational difficulties experienced. This enabled revision and refinement of the intervention before it was tested for feasibility and acceptability (pilot-testing).

## Primary objectives of the evaluation

1. To identify operational issues encountered by the study participants through field-testing
2. To revise the intervention based on the findings from the field-testing
3. To evaluate the feasibility and acceptability of the intervention among the stroke survivors and their caregivers through pilot-testing.

## Methods

### Mixed-methods research design

This study applied mixed research methods in order to collect more comprehensive evidence regarding the research question. The mixed-methods approach was specifically chosen because it is known to encourage the use of multiple worldviews and is a pragmatic approach to research pertaining to development of complex interventions.^11^

### Participant selection and recruitment

Only one hospital (TS Srinivasan Institute of Neurological Sciences, Voluntary Health Services (VHS) Multispecialty Hospital, Chennai) provided permission to recruit participants. The newly developed ‘Care for Stroke’ intervention was evaluated with a sample of 60 adult stroke survivors and their caregivers living in Chennai, South India (30 pairs of stroke survivors and their caregivers for field-testing and 30 pairs for pilot-testing). All were previously treated for their stroke at the VHS Hospital, which has an admission rate of three to four stroke patients per week. Given the hospital admission rate and the time that was available within the PhD project, we were able to recruit only 30 pairs of participants for field-testing and 30 pairs for pilot-testing.

Study participants were purposively selected from the hospital records and invited to the hospital for follow-up. Contact details of participants were retrieved from their hospital records. During the follow-up consultation, the stroke survivor was assessed for their eligibility to participate in the study by a neurologist. If the participant was determined to be eligible, they were provided with a detailed background of the study and its purpose by the investigator (KS). Informed written consent was obtained from those who agreed to participate in the study.

### Inclusion criteria

- Adults (aged ≥18 years)
- Recent diagnosis of first-ever stroke as defined by the WHO^12^ within 3–6 weeks of the recruitment
- Severity of stroke: minor and moderate (score 1–15, according to the NIH Stroke Scale^13^ ^14^).
- Stroke survivor medically stable (reaching a point in medical treatment where life-threatening problems following stroke have been brought under control)
- Post-stroke functional status of the stroke survivor: requiring assistance of at least one person to perform daily activities such as transfers, self-care and mobility (scoring less than the maximum score obtainable in one or more components of the Barthel Index (BI)^15^)
- Stroke survivor residing with a primary caregiver (family member) at home.

### Exclusion criteria

- NIH Stroke Scale score >15
- Severe cognitive difficulties (scoring >1 in Orientation, Executive function, Inattention and Language components of the NIH Stroke Scale for cognition)^16^
- Severe communication problem (scoring >1 in Dysarthria and Best Language component of the NIH Stroke Scale^13^ ^14^)
- Severe comorbidities (severe psychiatric illness, hearing loss, vision loss)
- Stroke survivor functionally dependent because of other pre-existing conditions (eg, amputation, fracture, dementia)
- Stroke survivor without a primary caregiver
- Stroke survivor unwilling/unable to adhere to the study protocol
- Did not meet the training requirements regarding operation of a smartphone

### About the intervention

The ‘Care for Stroke’ intervention was delivered through a smartphone and included information about stroke and the ways to manage post-stroke disabilities. This was provided through text and videos in the local Tamil language. The intervention is web-based and hence requires an internet connection. It includes modules on information about stroke, home-based exercises, functional skills training, activities of daily living, and assistive devices. Further details about the intervention have been described previously^17^ and as an online supplementary file 1.

### Training and administration of the intervention

The educational intervention was preloaded on to the smartphone. The stroke survivor and their caregiver received 20–30 min of training from the investigator (KS) on access and use of the intervention via the smartphone. Participants were then provided with a smartphone preloaded with the ‘Care for Stroke’ intervention (ie, a smartphone along with the intervention loaded on to it) and asked to try it out on their own. Three or more errorless attempts to retrieve the required part of the intervention from the smartphone were considered successful training.

Participants were asked to use this intervention at home for 2 weeks during the field-testing phase and for 4 weeks during the pilot-testing phase. The caregivers of stroke survivors selected for this study were asked to support the stroke survivors in accessing the intervention from the smartphone as and when required.

### Direct observation and interviews during field-testing

Utilisation of the smartphone-enabled intervention and the support provided by the caregivers to the stroke survivors was assessed by the investigator (KS). Direct participant observation (with observation checklist) and short unstructured interviews related to the objectives of the field-testing were carried out at each participant's home during this phase. Key issues assessed included:

- Relevance and comprehensibility
- Operational difficulties and user-friendliness
- Technical issues
- Training needs

### Assessment of feasibility and acceptability during pilot-testing

Feasibility and acceptability of the intervention was assessed primarily through a semistructured questionnaire administered to stroke survivors and primary caregivers. The majority of questions in the questionnaire were related to satisfaction and patient experience. The questionnaire predominantly included closed-ended questions with ordered (Likert scale) responses (see online supplementary file 2). The frequency of each response was calculated separately for each question in the questionnaire. The questionnaire schedule was developed, translated and pilot-tested before it was administered. In addition to this, participants were also asked specific open-ended questions related to the objectives of the pilot-testing. Participants’ responses to the questions were transcribed verbatim and translated into English. Transcribed data were then analysed using the framework approach.^18^

### Assessment of clinical outcomes

Independence in activities of daily living was assessed using the BI,^15^ and disability was assessed using the Modified Rankin Scale (MRS).^19^ The investigator (KS) carried out this assessment to investigate the feasibility of using these clinical outcome measures in a future larger trial of the intervention.

### Analysis of clinical outcome measures

Pre-intervention and post-intervention scores for the BI and MRS were analysed using the paired Student t test method.

## Results of the field-testing

The demographic and clinical characteristics of the stroke survivors and their caregivers are described in table 1.

**Table 1:** Demographic and clinical characteristics of the stroke survivors and caregivers in field-testing and pilot-testing

Characteristic	Participants in field-testing	Participants in pilot-testing	Statistical difference between the groups (p value)
***Stroke survivors***			
Gender			
Male	20 (67%)	18 (60%)	0.59
Female	10 (33%)	12 (40%)	0.59

Age (years)	54.2 (14.7)	57.9 (11.2)	0.27

Education, primary school or higher	24 (80%)	26 (87%)	0.49

Currently married	27 (90%)	30 (100%)	0.08

Working before stroke	26 (87%)	16 (53%)	0.0048*

Currently working	15 (50%)	3 (10%)	0.0007*

Stroke type			
Ischaemic	27 (90%)	24 (80%)	0.28
Haemorrhagic	3 (10%)	6 (20%)	0.28

Stroke severity			
Minor	12 (40%)	8 (27%)	0.27
Moderate	18 (60%)	22 (73%)	0.27

Affected side			
Right	18 (60%)	18 (60%)	1.00
Left	12 (40%)	11 (37%)	0.79
Both	0 (0%)	1 (3%)	0.31

Level of dependence			
Independent-personal care	15 (50%)	7 (23%)	0.032*
One-person assistance	15 (50%)	23 (77%)	0.032*

Receiving physiotherapy	8 (27%)	8 (27%)	1.00

Using mobility aids	9 (30%)	6 (20%)	0.37

Smartphone user	11 (37%)	3 (10%)	0.015*

***Caregivers***			
Gender			
Male	15 (50%)	11 (37%)	0.30
Female	15 (50%)	19 (63%)	0.30

Age (years)	31.6 (7.66)	39.5 (13.7)	0.008*

Education, primary school or higher	30 (100%)	29 (97%)	0.31

Employed	21 (70%)	27 (90%)	0.05

Primary caregiver	16 (53%)	25 (83%)	0.012*

Owns a smartphone	21 (70%)	21 (70%)	1.00

Smartphone user	28 (93%)	18 (60%)	0.012*

Values are mean (SD) or N (%).

*Significant difference between groups, p<0.05.

### Ability of participants to access the intervention from a smartphone

#### Stroke survivors

Among 30 stroke survivors selected for the field-testing, 37% (11 participants) had used a smartphone before their stroke. During the field-testing, seven stroke survivors (23%; six men and one woman) independently accessed the intervention through a smartphone. All remaining participants were helped by their caregivers to access the intervention—especially in operating the smartphone to access desired videos. Three stroke survivors (10%) used headphones to listen to the audio while watching the videos. Stroke survivors preferred to use their affected hand to hold or stabilise the smartphone and operate it using their unaffected hand. Most often, stroke survivors preferred to watch the video first, understand it and then practise the techniques shown at a later point.

#### Caregivers

Among the caregivers included in field-testing, 93% (n=28) were smartphone users before the intervention and 70% (n=21) owned a smartphone. None of the caregivers had difficulty in operating the smartphone and accessing the intervention. They generally helped the stroke survivors to access the intervention and directed them to watch inter-related videos.

### Technical/operational issues encountered by the participants during field-testing

Operational issues encountered by participants included:

1. Poor connectivity inside the home
2. Video-streaming delay because of low 3G data allowance
3. Low audio levels (eg, participant resided in a noisy area)
4. English version of the intervention not understandable, and Tamil version needed
5. Inability to access various web pages of the intervention by sliding the touchscreen on the smartphones
6. Inadequate clarity of the pictures.

In addition, five stroke survivors (17%) and 15 caregivers (50%) expressed that they required more indepth training and an operational manual to adequately learn and access the intervention from the smartphone.

### Revision and finalisation of the intervention

The findings from the field-testing were shared with an expert group consisting of professionals from various rehabilitation disciplines experienced in stroke rehabilitation. After receiving their feedback and advice, the preliminary field-tested version of the ‘Care for Stroke’ intervention was revised. All the operational issues identified during the field-testing (eg, the connectivity issues, poor audio/video quality, delayed video-streaming, language issues, touchscreen sliding functionality) were rectified by the technical consultants. This revised version of the intervention was once again shared with these expert group members for their review and approval for finalisation. The finalised version of the intervention was then used for pilot-testing.

## Results of the pilot-testing

The demographic and clinical characteristics of the stroke survivors and their caregivers are described in table 1.

### Feasibility for recruitment

Study recruitment took place from December 2014 to February 2015. We identified 46 stroke survivors from the hospital records, of whom 30 were recruited (cause of exclusion: death, 2; lack of contact details, 2; ineligible, 4; resided far from hospital, 4; refusal, 4).

### Feasibility for training and utilisation

Nearly 80% (n=24) of the stroke survivors required support from their caregivers to use the intervention, 13% (n=4) said that they could manage by themselves, and 3% (n=1) required additional training to access the intervention. In contrast, 77% (n=23) of the caregivers managed the application themselves, 13% (n=4) required support from other caregivers at home and 7% (n=2) required further training. Details of the training needs and pattern of utilisation by study participants are provided in table 2.

**Table 2:** Details of participant responses from the satisfaction survey (pilot-testing)

	Initial impression about the intervention					
Participants	Interesting	Encouraging	Motivating	Consoling	All	None
Stroke survivors	7 (23%)	3 (10%)	1 (3%)	17 (57%)	2 (7%)	0 (0%)
Caregivers	9 (30%)	6 (20%)	10 (33.3%)	4 (13.3%)	1 (3.3%)	0 (0%)
	**Need for training and support to access the intervention**					
	**Need support from others**	**Can manage myself**	**Need training**	**Need training and support from others**	**Not sure**	
Stroke survivors	24 (80%)	4 (13%)	1 (3%)	0 (0%)	1 (3%)	
Caregivers	4 (13%)	23 (77%)	2 (7%)	0 (0%)	1 (3%)	
	**Overall confidence to use the intervention**					
	**Definitely confident**	**Confident to a greater extent**	**Confident to some extent**	**Confident to a small extent**	**Not confident**	
Stroke survivors	3 (10%)	9 (30%)	17 (57%)	1 (3%)	0 (0%)	
Caregivers	17 (57%)	12 (40%)	1 (3%)	0 (0%)	0 (0%)	
	**Utilisation pattern of the intervention**					
	**More than once a week**	**Whenever possible**	**More than once a day**	**Whenever** **necessary**	**Did not use**	
Stroke survivors	15 (50%)	14 (47%)	1 (3%)	0 (0%)	0 (0%)	
Caregivers	14 (47%)	9 (30%)	2 (6%)	5 (17%)	0 (0%)	
	**Practising the skills learnt from the intervention**					
	**Always**	**Frequently**	**Occasionally**	**Rarely**	**Never**	
Stroke survivors	7 (23%)	16 (53%)	6 (20%)	1 (3%)	0 (0%)	
Caregivers	7 (23%)	15 (50%)	8 (27%)	0 (0%)	0 (0%)	
	**Overall usefulness of the intervention**					
	**Definitely useful**	**Useful to a greater extent**	**Useful to some extent**	**Useful to a** **small extent**	**Not useful**	
Stroke survivors	19 (63%)	9 (30%)	2 (7%)	0 (0%)	0 (0%)	
Caregivers	9 (30%)	20 (67%)	1 (3%)	0 (0%)	0 (0%)	
	**Overall likeableness of the intervention**					
	**Yes definitely**	**Yes to a greater extent**			**Yes to some extent**	
Stroke survivors	17 (57%)	12 (40%)			1 (3%)	
Caregivers	27 (90%)	3 (10%)			0 (0%)	
	**Overall rating for the smartphone-enabled intervention**					
	**Excellent**	**Very useful**			**Satisfactory**	
Stroke survivors	16 (53%)	13 (43%)			1 (3%)	
Caregivers	20 (67%)	10 (33%)			0 (0%)	
	**Overall rating for the usefulness of the intervention**					
	**Extremely useful**	**Very useful**			**Useful to an extent**	
Stroke survivors	11 (37%)	17 (57%)			2 (7%)	
Caregivers	16 (53%)	14 (47%)			0 (0%)	
	**Interesting sections of the intervention for the participants**					
	**All sections**	**Stroke information section**	**Stroke information and exercises sections**	**Stroke information and ADL sections**	**Stroke information and functional skills sections**	**Exercises section**
Stroke survivors	12 (40%)	7 (23%)	5 (17%)	4 (13%)	1 (3%)	1 (3%)
Caregivers	17 (57%)	5 (17%)	4 (13%)	2 (7%)	0 (0%)	2 (7%)

Values are N (%).

ADL, activities of daily living.

### Smartphone utilisation among study participants

Ninety per cent (n=27) of the stroke survivors had a smartphone at home, and over 40% (n=12) of them had either mobile or broadband internet connection at their home before the intervention. Only 23% (n=7) of the stroke survivors owned a smartphone themselves. Nearly three-quarters (70%, n=21) of primary caregivers owned a smartphone, and about 60% (n=18) of these used all the features of their smartphone. One family member with thorough knowledge and experience of using a smartphone was available at a minimum for each stroke survivor to help them use the intervention.

### Relevance of the intervention

All participants reported that the intervention videos related to ‘the information about stroke, activities of daily living and exercises’ were very relevant to their rehabilitation needs following stroke. Almost all (97%, n=29) of the stroke survivors felt that the intervention was most relevant to their current rehabilitation needs. Most of the carers (77%, n=23) reported that the intervention was definitely relevant to the needs of the stroke survivors.

Although 50% (n=15) of the stroke survivors included in the study were functionally independent, they still found the intervention relevant to them. All the participants found the ‘information about stroke’ section very relevant, especially in terms of gaining awareness about the warning signs of stroke, and knowledge about stroke, its impact and various aspects of recovery (table 2). The caregivers reported that they gained confidence and motivation to support the stroke survivor in their family after watching the videos.

### Comprehensibility of the intervention

When the study participants were asked about the overall comprehensibility of the intervention, 63% (n=19) of stroke survivors and 77% (n=23) of carers felt that the intervention was easily comprehensible. Participants attributed this to the people who acted in the videos and the language in which the audio descriptions were presented.

The stroke survivors and caregivers reported that they understood various sections of the intervention through the photographs in the application alone. None reported problems in either understanding the videos or the corresponding voiceovers. Participants stated that high-definition videos and simple language helped them comprehend the intervention at ease.

Stroke survivors reported enjoyment from learning about the ‘Dos and Don'ts’ after stroke and the ways to manage daily living. They explained that they understood the recovery process and the ways to prevent another stroke after watching the intervention videos.

One stroke survivor explained:

I was so depressed because of this problem. I did not know whether this could come back like heart attack. Watching the videos about risk factors was such a relief. Now I understood that, if I control my sugar and have a proper balanced diet, I can be away from another stroke

### User-friendliness of the smartphone-enabled intervention

The intervention was loaded on to a Micromax Canvas A102 Doodle3 Smartphone. This smartphone had configurations appropriate for accessing the intervention with good connectivity, streaming speed and picture clarity, and was relatively cheap. Other key aspects of user-friendliness of the intervention included:

1. Light weight of the smartphone (584 g)
2. Wide screen of the smartphone (7 inches)
3. Video/picture quality and detailing (high definition)
4. Streaming speed (on demand—content delivery network (CDN))
5. Application design and access features (based on the needs expressed by the stroke survivors)

A stroke survivor reported:

It's good that this is in a video format—It would be very difficult for me to read or understand formal Tamil dialects with the problems in my eyes. I always like to watch TV and hence I quite like the idea of teaching us ‘what to do’ through videos. Compared to reading from a book, this is not so boring as well

### Usefulness of the intervention videos

Fifty seven percent (n=17) of stroke survivors and 47% (n=14) of carers reported that the intervention was very useful to them. The overall rating that the participants provided for the usefulness of the intervention is presented in table 2. Stroke survivors explained that the video format of the intervention was very motivating. They felt that the intervention provided very useful information about their problem, the causes of their stroke, and the ways to manage their recovery independently. A stroke survivor who was unable to transfer or walk without support said:

I can now move from my bed to chair with some support from my sister. I am very happy to have achieved this. I saw the videos on ‘how to move from one place to another with support’ and I practiced it with my sister. Thank you for helping me with your videos. I am planning to learn more from it

Almost all stroke survivors (96%, n=29) felt that the intervention videos were self-explanatory. The carers explained that the stroke survivors were able to accept the importance of engaging in their daily living tasks and becoming as independent as possible in their lives.

### Acceptability of the intervention

Two key features of the intervention that were most strongly valued by the majority of study participants were:

1. The Tamil audio descriptions of the intervention (local language)
2. The content of the intervention, especially the exercises and daily living task sections, explained through demonstration by individuals who resembled stroke patients from Tamilnadu.

A stroke survivor explained:

I didn't know that something like this is available in Chennai, I thought all these were in foreign countries. My son showed me some videos where doctors are speaking in English and I could not understand much. But I was able to understand many things from these videos on the phone—it was in Tamil so it was very easy

Stroke survivors expressed that they were motivated and encouraged to see the actual performance of daily living tasks using one-handed techniques by someone like them in the videos.

A stroke survivor reported:

I am surprised that a person with stroke can do things by himself with the strong hand. It's eye opening. I felt, why can't I try. I am now trying some of the tasks that I saw from the videos, especially to use my hand to eat and dress myself.

### Acceptability of the smartphone-enabled application

When the study participants were asked about the acceptability of the intervention, more than half of stroke survivors (57%, n=17) and almost all carers (90%, n=27) reported that the intervention was definitely acceptable. Overall, 40% (n=12) of the stroke survivors and 10% (n=3) of the carers felt that the intervention was acceptable to a greater extent (table 2).

Stroke survivors found the portability of the intervention very useful to them, as they were able to comfortably watch the intervention videos anywhere they wanted. Stroke survivors also said that portability was very helpful in allowing them to watch the intervention privately (at home or elsewhere) without disturbing others and without feeling shy about the discreet content.

It's a big family—we are nine people in a single home and one TV for all of us. The TV room will be busy all the time with lots of family members. This was one important reason why I prefer the smartphone instead of a DVD. I take this to any room or even my workplace and watch, it's convenient to carry and comfortable to watch—no one knows what I am watching. Otherwise people will feel pity about my situation

Caregivers reported that the smartphone required very minimal physical effort in terms of carrying or operating it.

A caregiver explained:

To get up from your place, go near TV to switch on, find the remote, give connections etc. It requires lots of work. I have to walk, bend and lift. I can't do all this with my own problems—this arthritis. This smartphone that you gave is a nice choice. Nothing other than movement of fingers to touch the screen is required. My wife watched it even when she was in bed sometimes.

Caregivers also appreciated the size of the smartphone screen, which was big enough to watch the videos comfortably without straining their eyes. They expressed that they were able to access the intervention from their own smartphone.

Caregivers found the repeatability of the intervention through simple touch and slide options very comfortable, especially in helping stroke survivors to remember important information from the intervention and to reinforce the importance of recovery. Caregivers also appreciated the design of the application and the ability to share the intervention videos with others globally.

A caregiver said:

My daughter, who lives in Singapore, wanted to know what this phone thing is all about. So we shared the details with her and asked her to watch it. Next day she called us and enquired whether we are watching it or not and she calls every day to find out what we watched.

### Overall rating for the intervention

Fifty three percent (n=16) of stroke survivors and 67% (n=20) of carers rated ‘Care for Stroke' as excellent. The remaining participants rated the intervention as very useful (table 2).

### Suggestions by participants

A major concern voiced by several participants (n=6) was internet connectivity, since intervention videos were streamed online through the web-based application service. These six participants were living in remote locations (outskirts of the city) with very poor connectivity. Participants with a broadband internet connection did not report any concerns about connectivity and online streaming issues. Two participants (7%) felt that the intervention could have been provided for longer, while several others reported that the intervention should have been provided when they were first hospitalised for stroke. Five participants (n=17%) requested a follow-up home visit by a member of the hospital team to reassess their recovery following stroke. Seven participants (n=23%) suggested that this intervention should be provided to every stroke survivor in every hospital and also to the public to prevent further strokes and its recurrence.

## Clinical outcomes

Results from the analysis of clinical outcomes showed statistically significant improvement in the scores of BI and MRS between before and after the intervention period (table 3).

**Table 3:** Details from the analysis of the outcome measures (pilot-testing)

Outcome	Pre-intervention (baseline)	Post-intervention (end point)	Mean difference with 95% CI	Test for overall change in scores
Barthel Index	57.8 (26.6)	70 (25.8)	−12.2 (−15.3 to −9.0)	−7.86 p<0.00001*
Modified Rankin Scale	3.2 (0.8)	2.7 (1.1)	0.5 (0.3 to 0.7)	5.75 p<0.00001*

Values are mean (SD).

*Significant difference, p<0.05.

## Discussion

The evaluation revealed that there was a minimum of one smartphone user and one smartphone in every participant's family. This indicates the availability and degree of smartphone penetration in a city like Chennai, which makes it potentially feasible for the smartphone-enabled carer-supported ‘Care for Stroke’ intervention to be widely used for provision of rehabilitation services in the future. The intervention was also found to be highly relevant, easily comprehensible, useful, likeable and satisfying to a greater extent. This implies a high level of acceptability of the intervention among the study participants. Given the lack of availability and accessibility of this kind of informational or educational intervention in India, ‘Care for Stroke’ fulfilled an important need among its users.

More than half of the stroke survivors were confident only to some extent in using the intervention, while this proportion was much lower among the carers. This in turn could explain why 20% (n=6) of stroke survivors and 25% (n=8) of primary carers used the intervention only occasionally and why two stroke survivors reported that the intervention was useful only to some extent. This may point to the need for more training for the stroke survivors in the use of the intervention.

Although the results from assessment of clinical outcomes were statistically significant, the amount of clinical gains obtained by the stroke survivors during the intervention period was relatively small^20^. Furthermore, since there was no control group, we could not attribute the improvement to the intervention. Given the clinical significance and the small sample size in the pilot-testing, the statistical results obtained from the outcome measures have to be carefully interpreted^21^. However, the objective of using these clinical outcome measures was to look at the feasibility of their use in future trials of the intervention where a control group would be used to help attribute cause of improvement in clinical outcomes. Despite a short intervention period (4 weeks), these clinical outcome measures were able to detect statistically significant differences, thus establishing their feasibility for use in future clinical trials and effectiveness evaluations of the ‘Care for Stroke’ intervention.

Field-testing of the intervention helped the investigators to address key operational uncertainties that could have affected feasibility and acceptability. It also provided an opportunity to review and revise the intervention before it was pilot-tested. Pilot-testing of the intervention before its effectiveness evaluation assisted investigators to understand the factors that could affect feasibility and acceptability of the intervention. It provided valuable information that could be used to plan and organise rigorous effectiveness evaluation of the intervention in the future. A phased approach to the development of the intervention facilitated provision of proper consideration to the practical aspects of evaluation, providing assurance that the intervention could be delivered as intended in the future.^22^

Accommodating multiple centres from the same geographical location for recruitment of participants for future studies could hasten the process of participant recruitment and thereby the evaluation process. Future studies could broaden the criteria for participant inclusion to more easily achieve the desired sample size and also to stratify the effects of the intervention by different subgroups of stroke survivors.

## Conclusion

Evaluation of the ‘Care for Stroke’ intervention establishes its feasibility in an Indian context and its acceptability among the study stroke survivors and their caregivers. This makes it possible for us to affirm that provision of a smartphone-enabled, carer-supported educational intervention for management of post-stroke disabilities could be a potential strategy to meet the growing need for stroke rehabilitation services in settings where rehabilitation resources are very limited. Adoption and modification of the ‘Care for Stroke’ intervention, with due attention to the cultural aspects of the target population, could potentially help to bridge the gaps in access to stroke rehabilitation services not just in India but also in other low-resourced countries where the rehabilitation needs of stroke survivors are substantial.

## References

[1] Mackay J, Mensah G The atlas of heart disease and stroke. Geneva, Switzerland: WHO 2004 http://www.who.int/cardiovascular_diseases/en/cvd_atlas_15_burden_stroke.pdf (accessed 5 Apr 2011).

[2] Disability and rehabilitation action plan 2006–2011. Geneva, Switzerland: WHO, 2005 http://www.who.int/disabilities/publications/dar_action_plan_2006to2011.pdf (accessed 9 Jun 2015).

[3] World report on disability. Geneva, Switzerland: WHO, 2011 http://www.who.int/disabilities/world_report/2011/report/en/ (accessed 5 Apr 2015).

[4] Ferri CP, Schoenborn C, Kaira L Prevalence of stroke and related burden among older people living in Latin America, India and China. J Neurol Neurosurg Psychiatr 2011;82:1074–82. 10.1136/jnnp.2010.234153 PMC3171978 21402745

[5] Mechael PN The case for mHealth in developing countries. Innovations 2009;4:103–18.

[6] Ryu S Book review: mHealth: new horizons for health through mobile technologies: based on the findings of the second global survey on eHealth (global observatory for eHealth series, volume 3). Healthc Inform Res 2012;18:231–3. 10.4258/hir.2012.18.3.231

[7] Craig P, Petticrew M, Developing and evaluating complex interventions: reflections on the 2008 MRC guidance. Int J Nurs Stud 2013;50:585–7. 23159017 10.1016/j.ijnurstu.2012.09.009

[8] Craig P Foreword. In: Richards D, Rahm Hallberg I, eds. Complex interventions in health: an overview of research methods. Routledge, 2015:15–17.

[9] Tugnawat D, Thippaiah A, Kumar S Rehabilitation needs assessment among stroke patients in tertiary hospital of Andhra Pradesh. Indian J Phys Ther 2015;3: 38–42.

[10] Sureshkumar K, Murthy GVS, Kinra S Development and evaluation of a smartphone-enabled carer-supported educational intervention for management of disabilities following stroke in India—Protocol for the research study. BMJ Innovations 2015;1:117–26. 10.1136/bmjinnov-2015-000042 PMC4687506 26751379

[11] Craig P, Cooper C, Gunnell D Using natural experiments to evaluate population health interventions: new Medical Research Council guidance. J Epidemiol Community Health 2012;66:1182–6. 10.1136/jech-2011-200375 22577181 PMC3796763

[12] WHO MONICA Project Investigators. The World Health Organization MONICA Project (Monitoring trends and determinants in cardiovascular disease). J Clin Epidemiol 1988;41:105–14. 3335877 10.1016/0895-4356(88)90084-4

[13] Department of Health and Human Services. NIH stroke scale training, Part 2. Basic instruction. The National Institute of Neurological Disorders and Stroke (NINDS), 2010.

[14] Ver Hage. The NIH stroke scale: a window into neurological status. Nursing Spectrum (Greater Chicago) 2011;24:44–9.

[15] Mahoney F, Barthel D Functional evaluation: the Barthel Index. Md State Med J 1965;14:61–5. 14258950

[16] Cumming TB, Blomstrand C, Bernhardt J The NIH stroke scale can establish cognitive function after stroke. Cerebrovasc Dis 2010;30:7–14. 10.1159/000313438 20424439

[17] Sureshkumar K, Murthy GV, Munuswamy S ‘Care for Stroke’ a web-based, Smartphone-enabled educational intervention for management of physical disabilities following stroke: Feasibility in the Indian context. BMJ Innovations 2015;1:127–136. 10.1136/bmjinnov-2015-000056 PMC4516008 26246902

[18] Gale NK, Heath G, Cameron E Using the framework method for the analysis of qualitative data in multi-disciplinary health research. BMC Med Res Methodol 2013;13:117 10.1186/1471-2288-13-117 24047204 PMC3848812

[19] Van Swieten JC, Koudstaal PJ, Visser MC Interobserver agreement for the assessment of handicap in stroke patients. Stroke 1988;19:604–7. 10.1161/01.STR.19.5.604 3363593

[20] Philip S Clinical significance versus statistical significance. BMJ 2014;348:g2130 10.1136/bmj.g2130

[21] Philip S The importance of statistical power. BMJ 2013;347:f6282 10.1136/bmj.f6282

[22] Moore GF, Audrey S, Barker M Process evaluation of complex interventions: medical research council guidance. BMJ 2015;350:h1258 10.1136/bmj.h1258 25791983 PMC4366184

